# “Alas poor Yorick”: What retrospective analysis of canine skulls can tell us about the impact of environmental factors on health

**DOI:** 10.4236/ojas.2013.33A002

**Published:** 2013-07-17

**Authors:** Luciano O. Valenzuela, Kevin Chase, Lawrence McGill, Shawn Miller, Mark Nielsen, Karl G. Lark

**Affiliations:** 1 Department of Biology, University of Utah, Salt Lake City, USA; 2 Laboratorio de Ecología Evolutiva Humana, CONICET, Universidad Centro de la Provincia de Buenos Aires, Buenos Aires, Argentina; 3 ARUP, Animal Reference Pathology Division, Salt Lake City, USA

**Keywords:** Portuguese Water Dog, Necropsy, Histopathology, Exercise, Nutrition, Stable Isotope, Skull, Dentine, Collagen

## Abstract

Necropsies and extensive histological evaluation for clinical and sub-clinical disease of approximately three hundred Portuguese Water dogs are available as part of an ongoing study to assess their state of health at end of life. Throughout life these dogs enjoyed a variety of lifestyles and environments. Here we carry out retrospective quantitative assessments of life-time dietary input and physical activity for each dog. To do this, collagens from skull vault bone and from dentine have been analyzed for ratios of stable isotopes to determine differences in diet that individual dogs experienced during late or early life respectively. Robustness of skull bone (weight/unit of skull size) was used as a relative indicator of the amount of physical activity experienced during a dog’s lifetime. These environmental parameters were correlated with the frequency and severity of specific disease processes determined at necropsy. Both measures were shown to exert significant low-level (r < 25%) differential effects on specific diseases. The value of retrospective analysis of environmental influences is discussed.

## 1. INTRODUCTION

Complex disease phenotypes are increasingly becoming the subject of whole genome analysis in well-structured populations. Genetic analysis of complex diseases is often constrained by the influence of environmental factors that may obscure genetic signals or play an important role in conditioning the disease process (e.g. carcinogens).

Retrospective, quantitative, assessment of environmental factors is, therefore, an important adjunct to quantitative genetic analysis.

The dog (*C. familiaris*) presents an excellent model system with which to analyze this concept in detail. This has been made possible by the availability of ca. 300 Portuguese Water dogs (PW dogs) that have been necropsied for state of health at end of life [1]. These dogs, raised by different owners located throughout the continental United States and Canada have experienced a variety of life styles (exercise and diet) primarily determined by the breeders in whose kennels they originated and by the subsequent owners with whom they lived. In this paper, we use data from the skulls of necropsied dogs to retrospectively examine environmental influences on the frequency and severity of disease. For this purpose we have analyzed the correlation of two sets of metrics—skull robustness (weight/unit of skull size) and stable isotope ratios of skull and dentine collagens—with the frequency or severity of histopathological changes observed at necropsy.

### 

#### The impact of exercise on health

Lieberman *et al*. [2] used pigs run on tread mills to demonstrate that skull robustness increased with increasing amounts of exercise. They interpreted their results to suggest that varying amounts of exercise effected bone growth in all parts of the mammalian skeleton. PW dogs exhibit a wide range of variation in skull weights between skulls of comparable size. We have therefore used the robusticity of the skull as a method to retrospectively evaluate exercise during a dog’s life.

#### The impact of nutrition on health

We have extracted nutritional data retrospectively by analyzing the stable isotope (non-radioactive) ratios of carbon, nitrogen and sulfur found in dentine collagen (laid down early in life) and from skull vault collagen (laid down and recycled throughout life [3]). Ratios of stable isotopes have already proved valuable for inferring diets of primitive peoples and extant animal species, as well as geographical locations of habitats and migrations of marine and terrestrial animals. Ratios of ^13^C/^12^C, ^15^N/^14^N and ^34^S/^32^S are determined by the primary source of these elements. Carbon isotope ratios (*δ*^13^C) reflect the isotope ratios of the primary dietary carbon source and indicate the proportion of C_3_ plants (lower *δ*^13^C values in e.g., temperate grasses, fruits, vegetables, wheat, soybean) or C_4_ plants (higher values in e.g., tropical grasses, corn, sugar cane) consumed directly or indirectly as a component in animal food. Nitrogen isotope ratios (*δ*^15^N) reflect the original nitrogen source (low values for N fixation for plants grown on synthetic fertilizers, and higher values for plants using other natural reactive species such as natural nitrate. Sulfur isotope ratios (*δ*^34^S) have primarily been used to differentiate diets of terrestrial origin (lower *δ*^34^S values) from marine-derived ones (higher values). Subsequently, discrimination between isotopes (primarily for nitrogen) continues to occur at each metabolic cycle during the rise in trophic level (by soil microbes, during plant metabolism and within the animals that feed on these plants). Thus the ultimate isotope ratio of a consumer will be determined in large part by the original nutrient source of the food ingested and the trophic level of the food ingested [4–6]. This will vary according to diet and to the location of the raw materials used in preparing that diet, for example the use of marine as contrasted with terrestrial sources of protein. Therefore, measuring stable carbon, nitrogen and sulfur isotopes in collagenous tissues provides an integrated and retrospective view of protein sources being consumed.

In the material presented below, we correlate variation in skull robustness and stable isotopic signatures with specific histopathologies found at necropsy. This retrospective evaluation of variation in surrogates for exercise and diet suggests that specific effects of these environmental factors on age related diseases occur but are of relatively low magnitude.

## 2. MATERIALS AND METHODS

### 2.1. Necropsy Data

Cadavers sent to the University of Utah by owners were necropsied as described previously [1]. Anatomical and pathological evaluation included 53 whole body or organ weights or dimensions, and one-to-several histological studies of each of 27 tissues. Additional samples were collected where gross lesions (e.g. tumors) were visible in non-designated tissues. Common histological observations were scored for each tissue on a severity scale of 0 (none), 1 (mild), 2 (moderate), 3 (severe). Summary scores for pathologies observed in multiple tissues (e.g. Fibrosis) were calculated for each dog by averaging the severity score over all affected tissues.

### 2.2. Skull “Robustness” Data

Weights of cleaned skulls were measured without the mandible. 48 standardized points were measured using a 3D digitizer. All pairwise distances between these points were calculated and used for principal component analysis. The first principal component of these distances, explaining 64% of the total variation, was used as a measure of skull size. Skull weight was regressed onto skull size using the “lm” function of R [7]. The residuals from this regression were used as a measure of skull robustness.

### 2.3. Isotope Data

Samples of collagen were purified from bone (skull vault) and canine teeth (dentine) following standard procedures [8]. Briefly, the calcified bone matrix was dissolved with HCL and the residual collagen matrix subsequently purified and dried. Teeth were crushed into small pieces decalcified and purified in a similar manner to the bone sample, following standard protocols [2]. Dry material was separated for isotope analyses. Skull vault and dentine collagen samples from three dogs were analyzed for purity by mass spectrometry. All samples yielded peptide fingerprints identifying the protein samples as collagen.

#### Sample analyses

Collagen samples were analyzed using an isotope ratio mass spectrometer operated in continuous flow (CF-IRMS) mode in the Stable Isotope Ratio Facility for Environmental Research (SIRFER) at the University of Utah. For *δ*^13^C and *δ*^15^N analyses, 1 mg (± 10%) of material was used; for *δ*^34^S, 9 to 12 mg (±10%) were used. The collagen material was placed into small tin capsules that were then loaded into a zero-blank auto-sampler interfaced with an elemental analyzer (Carlo Erba) where they were combusted to produce N_2_ and CO_2_ or SO_2_. These gases were chromatographically separated and carried to the CF-IRMS (Finnigan MAT Delta S). All samples were analyzed alongside a set of internal laboratory reference materials that had been previously calibrated against international standards. Results for *δ*^13^C values are presented on the Vienna Pee Dee Belemnite (VPDB) scale, those for *δ*^15^N values on the AIR scale, and for *δ*^34^S values on the Vienna Canyon Diablo Troilite (VCDT) scale. The analytical precision (1), based on long-term measurements of internal laboratory reference materials for *δ*^13^C, *δ*^15^N and *δ*^34^S values, was 0.1‰, 0.2‰ and 0.4‰, respectively. Stable isotope values are reported using the standard *δ*-notation relative to an international standard in units “per mil” (‰) as follows: *δ* = 1000 (R_sample_/R_standard_ − 1), where R_sample_ and R_standard_ are the molar ratios of the heavy to light isotopes of the sample and standard, respectively.

### 2.4. Statistical Methods

#### Correlations and permutations

Pearson product correlations “r” between two trait vectors (t1 and t2) were calculated with the “cor (t1, t2, use = “pair”)” function of R [7]. Significance was established using 100,000 – 1,000,000 permutations where one trait was randomized with respect to the other. P-values are reported as the fraction of permutation trials for which the absolute value of r was greater than or equal to the non-permuted absolute value of r.

#### Heritability

Heritability was estimated using a generalized linear mixed model from the MCMCglmm package [9] of R [7]. The MCMCglmm function was run using the marker validated pedigree for the Portuguese Water dog population and the following settings: nitt = 1,000,000, thin = 195, burnin = 25,000.

## 3. RESULTS

### 3.1. Skull Robustness

**Figure 1:**
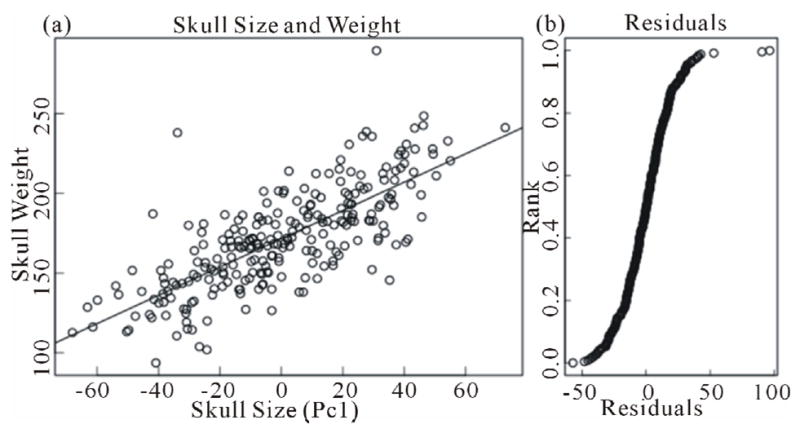
Correlation of skull weight with skull size. (a) Scatter graph comparing skull weights with skull size (PC1, see Methods). (b) Cumulative distribution of the residuals in (a).

Skull weights from PW dogs were regressed on skull size (Figure 1(a)), and values of residuals (robustness) determined (Figure 1(b)). Based on pedigree and molecular (DNA marker) relatedness of the dogs (see Methods) we have measured the heritability of this trait. Although the range of residual values is large, we were unable to establish heritability suggesting that heritability, if it exists, is less than 23%. Correlating residuals with specific pathologies suggested that specific correlations might exist, but that correlations were low (~20%) and therefore the amount of variation involved was small, ~5%. Although analysis of histopathological change was extensive (see Methods and [1]), only a few correlations were potentially significant. Table 1 presents the most significant correlations observed. Not unexpectedly, less robust skulls were correlated significantly with several pathologies. An unexpected result was the small, but significant correlation of more robust skulls with the frequency of sarcomas.

### 3.2. Stable Isotope Ratios

**Figure 2:**
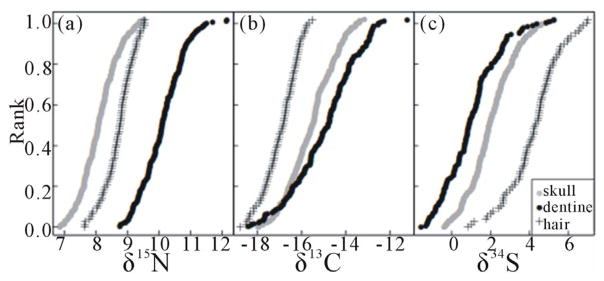
Cumulative distributions of discrimination values for *δ*^15^N, *δ*^13^C, or *δ*^34^S from skull-vault (SV) or dentine (DN) collagen (see Methods). Values for individual dogs are graphed as a function of their isotope ratio values: A. *δ*^15^N0/00; B. *δ*^13^C0/00; or C. *δ*^34^S0/00). Values for human hair [10,11] are shown for comparison.

**Figure 3:**
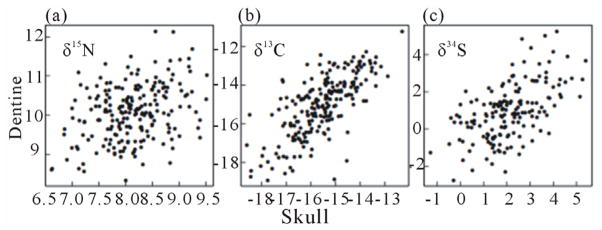
Scatter graphs comparing DN (ordinate) and SV (abcissa) discrimination values (*δ*) of individual dogs. (a) *δ*^15^N0/00: r^2^ = 0.42 (p < 10 – 5; 206 dogs); (b) *δ*^13^C0/00: r^2^ = 0.71 (p < 10 – 5; 206 dogs); (c) *δ*^34^S0/00: r^2^ = 0.56 (p = < 10 – 5; 163 dogs).

Figure 2 presents isotope ratios for C, N and S from PW dog collagen purified from dentine (DN) and skull vault (SV). For comparison SI analysis of human hair [10,11] is also shown. The range of values (C: 4‰; N: 2‰; S: 4‰) is similar for both DN and SV collagen, although the carbon and nitrogen values are on average at a lower trophic level in the SV collagen than in the DN collagen. In contrast Sulfur values were greater in SV than in DN collagen suggesting that sulfur and nitrogen differ in nutritional source and/or metabolic pathways between juveniles and adults. Nevertheless, there is a high correlation between the ratios found for dentine and skull: *δ*^15^N values r = 0.42; *δ*^13^C values r = 0.71; and *δ*^34^S values r = 0.59 (scatter graphs supporting correlation data are presented in Figure 3). These correlations suggest that nutritional sources (particularly for carbon) tend to remain the same throughout the lifetime of individual dogs. Since we know the relatedness (by pedigree) between dogs, we can calculate their consanguinity and estimate heritability of the values for discrimination presented in Figure 2. The estimated heritability is high for *δ*^13^C (SV, 0.55; DN, 0.68) and substantial for *δ*^15^N (SV, 0.38; DN, 0.39) and *δ*^34^S values (SV, 0.52; DN, 0.11). These cannot be measures of true heritability (e.g. additive genetic variation), because the range of values for *δ*13C (see Figure 2) far exceed the variation expected due to metabolism within a single trophic level, *i.e.* by an individual dog. More likely the high heritability estimate is due to a “Kennel effect” such that particular diets are associated with individual kennels that recommend the diet to subsequent owners. Thus the consanguinity resulting from breeding by a kennel is also associated with that kennel’s diet. (This would be consistent wit h the tendency for diets to persist during the lifetime of dogs resulting in the observed correlations between DN and SV ratios described above).

Figure 2 also displays results obtained for adult human hair. A large data set exists for isotope ratios of human hair, whereas relatively few samples of human collagen, from surgical samples, have been analyzed. We have therefore compared our dog data to data on human hair. Although there is a difference in the means of the two data sets, the variance is similar. The difference in the means is probably due to differences between collagen and hair, since such differences also have been observed between human collagen and human hair [12,13].

#### Correlation of Stable isotope ratios with organ metrics obtained at necropsy

We have estimated the impact of variation in isotope ratios on size metrics of organs and tissues obtained at necropsy from PW dogs. To do this, values of isotope ratios were correlated with metrics of various organs and tissues for individual dogs (weights or dimensions e.g. lengths of trachea or intestine) adjusted for differences in size and sex. (PW dog sizes can vary by somewhat more than two fold.) Body size was estimated as the first principle component (PC1) of the organ and tissue metrics. *δ*^15^N, *δ*^13^C, or *δ*^34^S values obtained from either SV or DN were not significantly correlated with any of the individual organ or tissue metrics. However, size (PC1) was significantly correlated with the *δ*^15^N values obtained from SV collagen (r = 0.198, p < 0.001). Isotope ratios obtained from DN collagen were not significantly correlated with size, nor were *δ*^34^S or *δ*^13^C values obtained from SV.

#### Correlation of Stable isotope ratios with pathologies determined at necropsy

We have analyzed the correlation between SI ratios obtained from skull (SV) or dentine (DN) with histopathologies observed in necropsied dogs.

Organs and tissues from necropsied animals were scored for 27 types of histopathological changes scored in multiple tissues or organs [1]. Table 2 presents those histopathological changes for which a correlation (r > 0.10) with an isotope ratio value was observed that were significantly different from zero (see Methods). There were no values of r appreciably greater than 20%. Correlations with age are also presented.

It can be seen that in many instances correlations were observed with isotope ratios obtained from DN but not SV collagens (*δ*^13^C values with cardiomyopathy, osteoporosis and glomerulosclerosis; *δ*^15^N values with atrophy, endocardiosis or glomerulosclerosis). The reverse was also observed, e.g. *δ*^15^N values with inflammatory bowel disease or lymphosarcoma.

For many pathologies, correlations observed with C ratios were not accompanied by significant correlations involving N or S ratios (or vice versa), whereas correlations to a particular pathology often involved both N and S ratios.

However in some cases, *δ*^15^N value correlations were observed without a similar correlation to *δ*^34^S values, (e.g. DN endocardiosis, SV inflammatory bowel disease); nor were significant correlations of *δ*^34^S values always accompanied by *δ*^15^N correlations (e.g. DN lymphosarcoma). It should be noted that isotope ratios from dentine collagen, laid down in early life, were often correlated with histopathologies observed most frequently in older animals (e.g. endocardiosis, glomerulosclerosis, or osteoporosis; see “Age” column).

## 4. DISCUSSION

The results point out the value of identifying associations between non-heritable phenotypes and disease based on the retrospective analysis of data inherent in the animal’s organs and tissues. The data presented above are derived from a population of genetically characterized PW dogs analyzed at necropsy for histological changes as part of an ongoing research project (The Georgie Project). The ultimate goal of that project is the genetic characterization of disease processes. The skulls of these dogs have served as a source of morphological (skull dimensions and weight) and biochemical (isotope ratio) data. Previous analysis of this necropsy database [1] found that individual dogs on necropsy exhibited multiple different histopathological changes (ranging from two to as many as 11 per dog with a mean for the population of seven different histopathologies/dog). Most of these are subclinical, with usually one or two associated with the death of the animal.

Lieberman [2] demonstrated that differences in skull robusticity could result from differences in the amount of exercise that an animal experiences and led him to propose that this was the basis for the difference in weight between skulls of primitive and modern man. Whether or not such a causative relationship is responsible for the differences in skull weights found in this PW dog population, the phenotype presents an opportunity to examine the role of environmental influences on the frequency of disease. We have been unable to demonstrate heritability for this phenotype in the PW dog population, in support of Lieberman’s hypothesis that variation in skull thickness is environmentally determined.

A similar retrospective process has allowed us to extract dietary information, in the form of *δ*^13^C, *δ*^15^N or *δ*^34^S values from dentine and skull vault collagens laid down early or later in life. The values in Figure 2 demonstrate several important aspects of diet in the PW dog population: 1) Like humans, PW dogs are omnivores; 2) The large range of values for *δ*^13^C is consistent with diets that utilize either Maize (C4) or Soybean (C3) as a basic source of caloric nutrition. (The separation of C3 and C4 plant values of *δ*^13^C is on average approximately 14‰ [14].) It should be remembered that the composition of these two nutrient sources is very different; 3) SV and DN collagens differ in the population profile of isotope ratios, most strikingly for *δ*^15^N. Thus they distinguish the product of diet/metabolism in early and later stages of life histories.

Although these data, do not inform us of the form in which a particular element is ingested (e.g. *δ*^13^C values as carbohydrate, fat or protein) they suggest valuable avenues for future investigations of the impact on specific diseases of diets during early and later life.

### 

#### Variation in Skull Weight

The observed variation in weight between skulls of comparable size (Figure 1) could be the result of exercise as suggested by the findings of Lieberman [2], changes in metabolism or diet prior to death, as well as other processes that affect accretion or loss of bone. Changes in skull weight are associated with small amounts of variation in the frequencies and/or severity of specific pathological processes (Table 1). Most notable was the positive correlation (heavier skulls) with sarcomas (mostly hemangio- and lymphosarcomas). It is possible that increased synthesis of bone may trigger the onset of these cancers or that an indirect effect of the cancer is to increase bone synthesis. Clearly this association warrants further investigation in other systems. Significant inverse correlations with specific histological changes also were found. These histopathologies appear to be unrelated to each other (e.g. IBD and atherosclerosis). These pathologies may not have resulted in death, but all were correlated with a relative loss of bone from the skull. Although a number of pathologies (e.g. many listed in Table 2) were not correlated with skull weight variation, lack of correlation with osteoporosis is of primary importance, because it suggests that variation in skull weight is not the result of aging. Indeed, we have observed only a slight correlation between variation in skull weight and age at necropsy, explaining at most 3% of the variation in skull robusticity.

#### Variation in isotope ratios

The data in Table 2 illustrate several important aspects of retrospective evaluation of diet. Although only small amounts of histological variation are associated with variation in isotope ratios, the specificity of those correlations is significant.

Four different pathologies were significantly associated with dentine collagen but not with skull vault collagen. Three of those associations—endocardiosis, glomerulosclerosis, and osteoporosis—involve diseases that are associated with aging. Thus the source of collagen implicates an early stage in life when diet influences the incidence of these later life pathologies. Dietary specificity also plays a role in these associations, since variation in *δ*^13^C but not in *δ*^15^N values influences the incidence of cardiomyopathy, glomerulosclerosis and osteoporosis, whereas *δ*^15^N influences the incidence of endocardiosis and IBD. The fact that correlations with *δ*^15^N are not always accompanied by correlations with *δ*^34^S values adds additional information. Correlations (e.g. with glomerulosclerosis) involving both *δ*^34^S and *δ*^13^C, but not *δ*^15^N values, were observed as well as correlations involving only *δ*^15^N values but not *δ*^34^S values (e.g. IBD or hemangiosarcoma). These imply dietary influences related to the origins of sulfur in the diet, as discussed by Richards *et al*. [5]. For example *δ*^34^S values vary between proteins from terrestrial and marine environments and can vary between locations within these ecosystems.

Finally, dietary correlations distinguish between lymphosarcoma and IBD on the one hand (inversely correlated with variation in *δ*^15^N) and hemangiosarcoma (positively correlated with *δ*^15^N) on the other. Additionally, *δ*^34^S values from dental collagen differentiated the influence of diet on the two cancers.

In summary, retrospective analysis of the influence of two environmental factors on disease has suggested differential roles of exercise and/or diet on specific diseases opening the way for more detailed investigations. It thus would appear profitable to develop additional retrospective assays for other environmental factors that may leave an imprint on an animal during its lifetime.

## Figures and Tables

**Table 1:** Correlation between skull weight/density and specific disease frequency and severity (see Methods).

histology trait	r
IBD	−0.26**
FIBROSIS of organs or tissues	−0.23**
ATROPHY of organs or tissues	−0.22**
SARCOMA	0.25**
ATHEROSCLEROSIS	−0.16*

Significance of correlation: <0.01 (*); < 0.0002 (**).

**Table 2:** Correlations (r) between histopathology of tissues at necropsy and isotope ratios. Correlations (r) significantly different from zero (see Methods) are shown in bold. Correlations significantly different between DN and SV (see Methods), are noted by an asterisk. The AGE column indicates the correlation between the histology score and age of death. (Positive values indicate that the pathological process is more pronounced in older dogs.)

	*δ*^13^C	*δ*^15^ N		*δ*^34^ S		AGE	

	DN (r)	SV (r)	DN (r)	SV (r)	DN (r)	SV (r)	
Atrophy of organs or tissues	0.19	0.18	−0.12	−0.04	−0.07	−0.04	−0.02
Carcinoma	0.01	−0.07	0.12	0.11	0.16	0.12	0.05
Cardiomyopathy	0.12	0.02	0.00	−0.06	−0.06	0.01	0.10
Endocardiosis	0.04	0.03	−0.17*	0.07	0.00	−0.02	0.18
Glomerulosclerosis	0.15	0.08	−0.12	0.00	0.18*	−0.07	0.43
Hemangiosarcoma	−0.08	−0.07	0.21	0.20	0.07	0.09	−0.01
Ibd (inflammatory bowel disease)	0.03	0.04	−0.04	−0.12*	0.04	−0.02	0.26
Lymphosarcoma	−0.07	0.00	−0.06	−0.11	−0.17	−0.15	−0.27
Osteoporosis	0.17*	0.01	−0.08	−0.02	0.06	0.09	
